# Epidemiology of Carbapenem Resistance among Multi-drug Resistant Enterobacteriaceae in Uganda

**DOI:** 10.9734/BMRJ/2015/17055

**Published:** 2015-05-02

**Authors:** Lucas M. Ampaire, Victoria Katawera, Dan Nyehangane, Yap Boum, Joel Bazira

**Affiliations:** 1 Department of Medical Laboratory Sciences, Mbarara University of Science and Technology, Mbarara, Uganda; 2 Department of Laboratory, Epicentre Mbarara Research Centre, Mbarara, Uganda; 3 Department of Microbiology and Immunology, Mbarara University of Science and Technology, Mbarara, Uganda

**Keywords:** Enterobacteriaceae, carbapenemase, multi-drug resistant, CPE, ESBL

## Abstract

**Background:**

Multi-drug resistant (MDR) Enterobacteriaceae are on the increase worldwide and their spread has become a global challenge. Escalating the challenge is the possibility that many of these are Carbapenemase-producing Enterobacteriaceae (CPE). This further complicates patient management. The magnitude of MDR-CPE in many developed settings has been reported, however, there is paucity of data from resource limited settings. We evaluated the epidemiology of MDR-CPE of clinical origin in South Western Uganda.

**Methods:**

From September 2013 to June 2014, all Enterobacteriaceae isolated from diverse specimens obtained from patients attending Mbarara Regional Referral Hospital, South-western Uganda, were screened for MDR in a laboratory-based cross sectional study. Isolates found to be MDR were screened for carbapenem susceptibility/resistance phenotypically by Kirby Bauer disc diffusion method following CLSI guidelines and genetically using the multiplex real-time Polymerase Chain Reaction (RT-PCR).

**Results:**

Of the 658 strains isolated, 183 (27.8%) were MDR and 68 (37.15%) of those MDR exhibited at least one form of carbapenem resistance with 23 (12.57%) and 56 (30.60%) isolates expressing phenotypic and genetic resistance, respectively. Eleven MDR-CPE (6.01%) isolates exhibited both phenotypic and genotypic resistance to carbapenems. Only *bla*_VIM_ and *bla*_OXA-48_ genes were detected among the genetically resistant isolates.

**Conclusion:**

The high prevalence of MDR-CPE calls for aggressive infection control and prevention strategies, including reinforcement of hand hygiene, using contact precautions and early detection of CPE through use of targeted surveillance and molecular techniques in resource limited settings.

## 1. INTRODUCTION

Carbapenems are crucial for the management of life-threatening infections [1] and are often the antimicrobials of last resort to treat infections due to extended-spectrum β-lactamase (ESBL)-or plasmid-mediated AmpC (pAmpC)-producing organisms of the Enterobacteriaceae family [1]. These pathogens are frequently also resistant to other antibiotic classes and are described as multi-drug resistant (MDR) [2–4].

Carbapenem-resistant Enterobacteriaceae (CRE), are a family of germs that are difficult to treat because they have high levels of resistance to antibiotics [5]. Resistance in these organisms is largely due to production of beta-lactamase enzymes [6] like *Klebsiella pneumoniae* carbapenemase (KPC), New Delhi Metallo-betalactamase (NDM) and Verona Integron-Mediated Metallo-β-lactamase (VIM), that break down carbapenems and make them ineffective [5].

Acquired class A (KPC), class B (IMP, VIM, NDM), or class D (OXA-48, OXA-181) carbapenemases are the most important determinants sustaining resistance to carbapenems [6–7]. The corresponding genes are mostly plasmid-located and associated with various mobile genetic structures (insertion sequences, integrons, transposons), further enhancing their spread.

Unfortunately, the prevalence of CPE has increased worldwide during the past 10 years, seriously compromising the therapeutic armamentarium [7–10]. There is paucity of data regarding their prevalence in Resource Limited Settings and as such, not much has been/ is being done to contain them. Although it is a requirement in hospital pharmacies to have a prescription note, in private pharmacies, patients access antibiotics freely over the counter without necessarily presenting a prescription note.

To ensure their containment, wide dissemination of information that will enable development of evidence-based strategies involving microbiologists, clinicians and other stakeholders is essential. Herein, we report the epidemiology of MDR Enterobacteriaceae isolates of clinical origin in a low income setting.

## 2. MATERIALS AND METHODS

We conducted a cross-sectional study at Mbarara Regional Referral Hospital (MRRH), Mbarara, Uganda, from September 2013 to June 2014. MRRH is the regional referral hospital in south Western Uganda. It provides public healthcare with general and teaching hospital facilities and has a capacity of more than 600 beds. The study was approved by the Faculty of Medicine Research and Ethics Committee (FREC), the Institutional Review Committee (IRC) of Mbarara University of Science and Technology and the Uganda National Council for Science and Technology.

Viable isolates of the Enterobacteriaceae family obtained from various clinical specimens of all patients attending MRRH during the study period were identified following standard microbiological procedures and then screened for phenotypic multi-drug resistance using Kirby-Bauer disc diffusion method following CLSI guidelines [11].

Isolates that screened positive for MDR were screened for carbapenem susceptibility/resistance phenotypically by Kirby Bauer disc diffusion method following CLSI guidelines [11]. Briefly, a 10 µg imipenem disc was placed on lawn culture of the isolate on Mueller Hinton agar and Phenotypic expression of a Carbapenemase was taken to be detected if the diameter of zone of inhibition was ≤19mm and, genetically using the multiplex real-time Polymerase Chain Reaction (RT-PCR) at Epicentre Mbarara Research Centre Laboratory. We used the QIAamp® DNA Min kit (QIAGEN, GmbH Ebensburg, German) for extraction and the Qiagen Multiplex PCR kit (QIAGEN, GmbH Ebensburg, German) for the amplification. PCR for the following carbapenemase genes *bla*_IMP_; *bla*_VIM_; *bla*_OXA-48_; *bla*_KPC_; was done as described previously [12–15].

Standardized controls were incorporated at all levels of analysis/ testing to rule out contamination and ensure good performance of the kits and processes. The control bacterial strains (*Klebsiella pneumoniae* 211 (T), *Klebsiella pneumoniae* 714, DSMZ *Escherichia coli* 9377 and *Escherichia coli* ATCC 25922) and DNA products were obtained from Institute of Microbiology, Gissen, Germany.

All the data were summarized as proportions. The primary outcome of interest was resistance to carbapenems. Prevalence ratios for the phenotypic and genetic characterization were obtained. Kappa statistics for the comparison between phenotypic and genotypic characterization were obtained. STATA, version 13 (StataCorp, College Station, Texas, USA) was used for all the analyses. A p-value ≤ 0.05 was considered to be statistically significant. The graphs and pie-charts were drawn using Microsoft Excel 2010.

## 3. RESULTS AND DISCUSSION

Of the 658 Enterobacteriaceae strains isolated, 183(27.8%), representing 22 different species of Enterobacteriaceae from a total of 11 types of clinical samples (Fig. 2), were found to be MDR and were screened for carbapenem resistance. *Escherichia coli* and *Klebsiella pneumoniae* were the most common isolated strains (54.1% & 18.6%, respectively) (Fig. 2). Of the 183 MDR isolates (Table 3), 68 (37.15%) exhibited at least one form of carbapenem resistance with 23 (12.5%, 95% CI: 7.7% – 17.4%) and 56 (30.6%, 95% CI: 23.8% – 37.3%) isolates expressing phenotypic and genetic resistance, respectively (Table 1, Table 2). Eleven (6.01%) isolates exhibited both phenotypic and genotypic resistance to carbapenems (Table 2). The isolates were recovered from both in-patients and out- patients.

The study obtained a prevalence of 30.6% of Carbapenem Resistance Determining Genes (CRDG) among multidrug resistant gram negative bacilli. This high prevalence is supported by previous reports in other parts of the world, 42.0% in Tanzania, between 31% and 55% in India and 43% in Tunisia [3,7–10,16], that necessitates immediate public concern and action.

Eleven isolates exhibited both genetic and phenotypic resistance. However, 12/23 phenotypically resistant isolates did not exhibit any of the tested genetic markers of resistance. This could be due to possession of other markers that were not tested in this study or new variants. Of the 56 isolates that expressed genetic markers of resistance, 45 isolates did not express phenotypic resistance. This is possibly due to possession, by some isolates, of silent genes that only exhibit phenotypic characteristics under conducive conditions.

The proportion of resistance by genetic characterization was significantly higher than that by phenotypic characterization (p= 0.0000, *K= 0.12*) (Table 2). Owing to this, molecular techniques enable early detection of resistance, better patient management and are a basis for early re-enforcement of infection control.

**Fig. 1:**
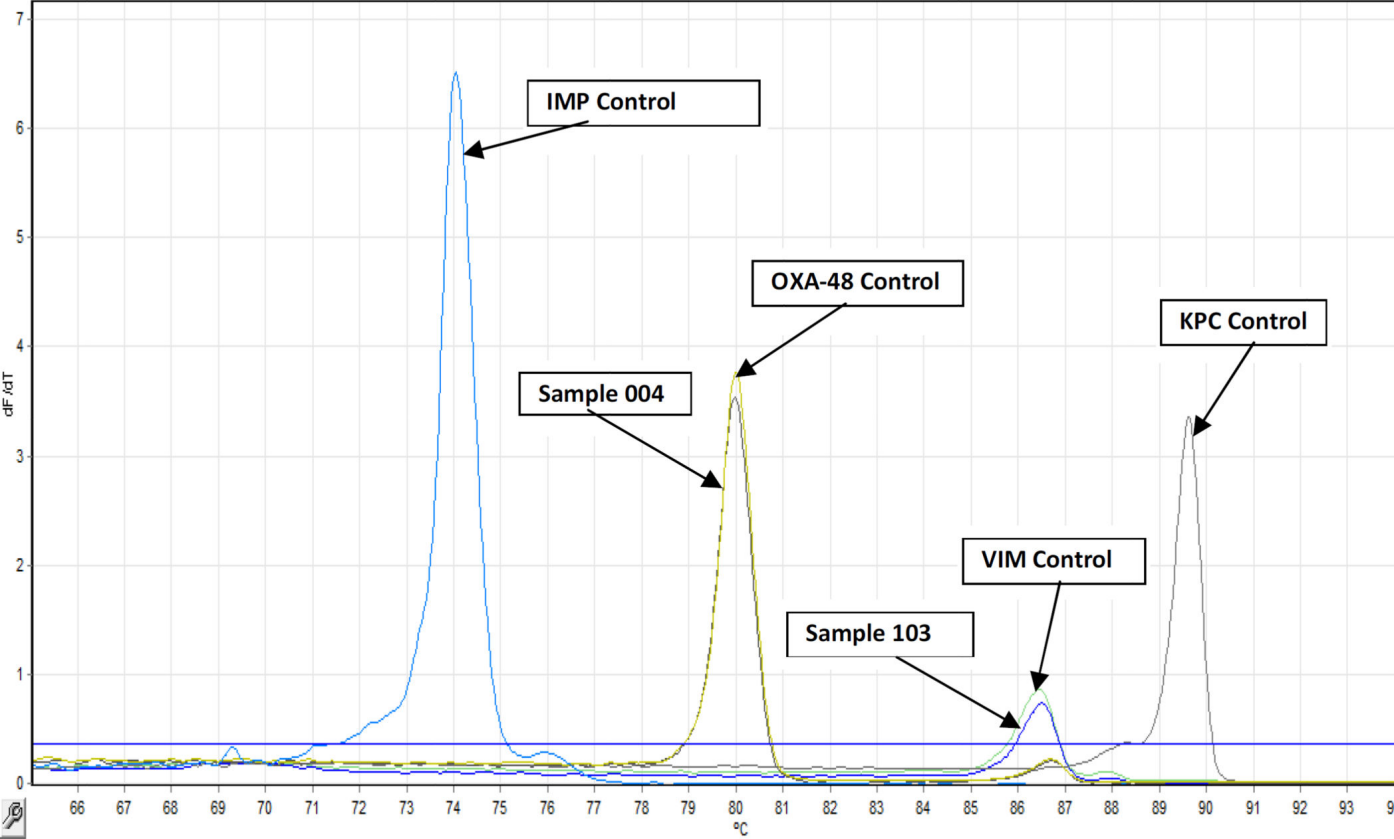
RT-PCR results An example of Results from Real –time multiplex PCR melting curves of amplicons generated by primers targeting four Carbapenemase types. The gene targets, from left to right, are as follows: bla IMP type (Tm 74.1°C), bla OXA-48 (Tm 80°C), blaVIM (86.6°C), blaKPC (89.7°C). Sample 004 and Sample 103 are clinical isolates that carried a carbapenem resistance determining gene

**Fig. 2:**
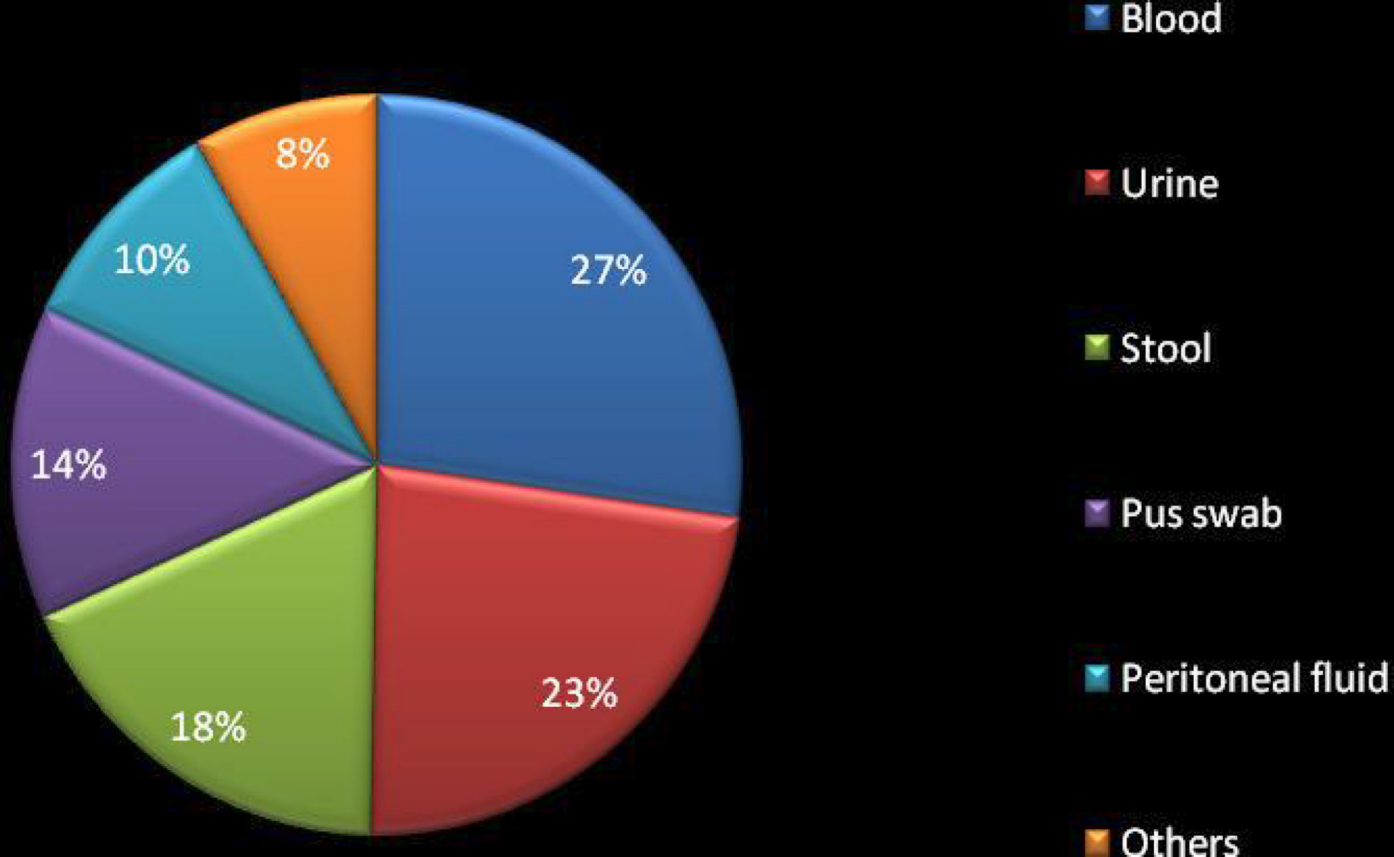
Distribution of clinical specimens used in the study (N=183) Others include; HVS, CSF, Pleural fluid, Sputum, Nasal swab

Of the most common carbapenem resistance determining genes (*bla*_VIM_, *bla*_KPC_, *bla*_OXA-48_, *bla*_IMP-A_, *bla*_IMP-B_ and *bla*_IMP-C_) tested, only bla_VIM_ and bla_OXA-48_ genes were detected (Fig. 1) among the genetically resistant isolates (Table 3). Genetic resistance in these isolates was therefore due to production of carbapenemases as previously reported [6,16]. VIM, which has been reported in several parts of the world [7,16] was the more frequent (43/56, 76.8%) carbapenemase identified in this population.

## 4. CONCLUSION AND RECOMMENDATIONS

The high prevalence of MDR-CPE calls for aggressive infection control and prevention strategies, including reinforcement of hand hygiene, using contact precautions and early detection of CPE through use of targeted surveillance and molecular techniques in resource limited settings. Also there is need to establish local antibiotic resistance surveillance teams so as to monitor and steward antibiotic use. There is need to study the clinical significance of PCR results in resource limited settings where phenotypic testing remains the most feasible method of resistance detection/prediction.

Further sequencing of these isolates could avail more information about genetic markers not considered in this study or new variants.

## Figures and Tables

**Table 1:** Phenotypic and genetic characterization

Variable	n (%)
**Phenotypic**	

Ceftriaxone	181 (98.91)
Ceftazidime	181 (98.91)
Cefot/cefuroxime	181 (98.91)
Ciprofloxacin	155 (84.70)
Imipenem	9 (4.92)
Total phenotypic	23 (12.57)

**Genotypic resistance**	

Genotypic markers of resistance	
VIM	43 (23.50)
OXA-48	13 (7.10)
Total genotypic resistance	56 (30.60)

**Table 2:** Phenotypic and genetic resistance

	Genetic characterization			Total
	
**Phenotypic** **characterization**	**Result**	**Resistant**	**Susceptible**	

	Resistant	11	12	23
	Susceptible	45	115	160
**Total**		56	127	183

K= 0.12, p-value =0.0276/ 0.02/ <0.05

**Table 3:** Distribution of different CRDG among the multi-drug resistant Gram negative bacteria

Isolate/organism	Carbapenem-resistance determining genes, No. (%)		Total detection
		
	VIM	OXA-48	
*Escherichia coli* (n=99)	20(46.5)	6(46.1)	26
*Klebsiella pneumoniae* (n=35)	17(39.5)	5(38.5)	22
*Proteus mirabilis* (n=10)	1(2.3)	1(7.7)	2
*Salmonella spp* (n=10)	3(7.1)		3
*Morganella morganii* (n=3)	1(2.3)		1
*Enterobacter sakazaki* (n=3)		1(7.7)	1
*Stenotrophomonas spp* (n=2)	1(2.3)		1
**Total**	**43 (100)**	**13(100)**	56
